# Preliminary Evaluation of a Nest Usage Sensor to Detect Double Nest Occupations of Laying Hens

**DOI:** 10.3390/s150202680

**Published:** 2015-01-26

**Authors:** Mauro Zaninelli, Annamaria Costa, Francesco Maria Tangorra, Luciana Rossi, Alessandro Agazzi, Giovanni Savoini

**Affiliations:** 1 Faculty of Agriculture, Università Telematica San Raffaele Roma, Via di Val Cannuta 247, 00166 Rome, Italy; 2 Department of Health, Animal Science and Food Safety (VESPA), Università Degli Studi di Milano, Via Celoria 10, 20133 Milan, Italy; E-Mails: annamaria.costa@unimi.it (A.C.); francesco.tangorra@unimi.it (F.M.T.); luciana.rossi@unimi.it (L.R.); alessandro.agazzi@unimi.it (A.A.); giovanni.savoini@unimi.it (G.S.)

**Keywords:** double nest occupation, imaging analysis, laying hens' performance and behavior

## Abstract

Conventional cage systems will be replaced by housing systems that allow hens to move freely. These systems may improve hens' welfare, but they lead to some disadvantages: disease, bone fractures, cannibalism, piling and lower egg production. New selection criteria for existing commercial strains should be identified considering individual data about laying performance and the behavior of hens. Many recording systems have been developed to collect these data. However, the management of double nest occupations remains critical for the correct egg-to-hen assignment. To limit such events, most systems adopt specific trap devices and additional mechanical components. Others, instead, only prevent these occurrences by narrowing the nest, without any detection and management. The aim of this study was to develop and test a nest usage “sensor”, based on imaging analysis, that is able to automatically detect a double nest occupation. Results showed that the developed sensor correctly identified the double nest occupation occurrences. Therefore, the imaging analysis resulted in being a useful solution that could simplify the nest construction for this type of recording system, allowing the collection of more precise and accurate data, since double nest occupations would be managed and the normal laying behavior of hens would not be discouraged by the presence of the trap devices.

## 1. Introduction

Council Directive 1999/74/EC reports that the welfare conditions of hens kept in current battery cages and in other breeding systems are inadequate. It bans conventional cages and demands the use of enriched cages in order to improve the hens' welfare [1]. The adoption of this law by the states within the European Union and the change of consumer preferences suggest that conventional cage systems in many commercial egg farms should be replaced by alternative housing systems in which hens are allowed to move freely in groups [2].

Although these alternative housing systems improve the welfare of the hens [3], they expose them to a greater opportunity for diseases, parasites, bone fractures and deleterious behaviors, such as cannibalism and piling [4]. Additionally, egg production is not as high or as stable as it is when produced with the usage of cages [5]. This effect may be largely attributed to the consumption of a balanced diet, which the cage environment guarantees [6]. In alterative housing systems, where a large group of hens is concentrated in a single area, the competition for space and resources can be observed [7]. In some cases, access to the feeders may be restricted due to social interactions [7]. In other cases, even though hens consume a similar amount of feed, a reduction in nutrient partitioning devoted to egg production may occur [6]. This reduction may be due to an increased demand of nutrients necessary to support the hens' behaviors in free-range environments [4,6]. The same effect is not observed in cage systems, because hens are exposed to lower stress levels, due to the highly regulated environments in which they are reared [6]. Therefore, no single housing system is ideal from a hen welfare perspective [4]. Although alternative housing systems increase behavioral opportunities, they introduce problems (e.g., diseases, unstable eggs production, *etc.*), and they give hens the opportunity to demonstrate behaviors that may be injurious to their welfare [4]. Breed selection may be the key factor for achieving both good welfare and high quality production outcomes in these alternative rearing systems. Existing commercial strains should be evaluated, and new selection criteria for future breeding programs should be developed on the basis of individual and accurate data about laying performance and the behavior of hens [7–10]. At the same time, these data could lead to the review of animal management and housing system designs/set-ups. Best practices could be developed in order to allow animals, also with different behavioral patterns, to demonstrate the necessary behaviors indicative of a positive welfare state [7]. Furthermore, a better coupling between the selected commercial strains and the housing systems used could be possible, with positive results also for the farmers. Up to now, the published data about the economic impact of implementing alternative management systems report that an increase of farm-level costs, of approximately 40% per dozen eggs, has been observed in case of shifting from conventional cages to free-range housing systems [11].

Many recording systems have been developed and tested [2,8,12–27] to collect data about laying performance and the behavior of hens. Among these studies, the most cited system is the funnel nest box (FNB) [8]. This system uses RFID technology to collect data about egg production and nest occupancy times for a single hen. Each hen carries a passive transponder tightened around its leg. An antenna, embedded in the nest floor, reads the transponder. The nest occupancy time is evaluated through repeated readings of the antenna. Combining the transponder ID, the egg sensor signal and the position of the egg in the egg collection tube, the system can figure out the “egg-to-hen” assignment. Another type of recording system was instead developed by Burel [12]. In this system, each hen carries two passive transponders, one injected into its neck and the other glued to its leg band. An antenna that surrounds the entrance of the nest reads the transponders. The transponder in the neck is used to record the hen's nest visit, while subsequent registrations from both transponders are used to record the number of times that the hen enters or exits the nest, on the basis of the transponders' reading sequence. The nest occupancy time is evaluated through the time-stamp difference between the entrance reading and the exit reading. The egg-to-hen assignment is obtained by combining the transponder ID, the egg sensor signal and the position of the egg in the collection tube, as in the FNB [8].

With these recording systems, the management of double nest occupations is critical. The egg-to-hen assignments can be wrong, or in systems like the FNB, data regarding the nest usage can be incomplete. This latter case may be due to the hen identification procedure, which is done by an antenna embedded in the floor. If two or more hens enter the nest and the transponders are in the same reading range, only one of these hens can be identified, while the others are missed. As a consequence, data recorded in this way may be wrong, or at least incomplete and not useful for evaluating the hens' behavior. For this reason, double nest occupations have to be limited and/or identified. In this regard, field trials on laying performance and the behavior of hens could be limited, and the selection of laying hens and/or improvements in the management and design/set-up of housing systems, in order to increase the hens' welfare, may not reach their target.

Most of these recording systems try to avoid double nest occupations through specific trap devices at the nest entrance. For instance, in the system developed by Marx [2], the trap device consists of two flaps (entry flap and blocking flap) arranged at an angle of 100° with respect to each other. When the nest is empty, the entry flap closes the nest entrance. To enter the nest, the hen pushes the entry flap forward and upward to the roof of the nest box. Then, the blocking flap comes down behind the hen, closing the entrance. As long as the hen remains in the nest box, the entrance is blocked, and no other hen may enter. In the FNB [8], a similar trap, based on a tilting floor, is placed at the entrance of the nest. When a hen enters the nest, the floor rotates. This rotation locks the trap and prevents other hens from entering while the nest box is occupied. Nevertheless, in these systems, a double nest occupation can surely occur, in most instances due to the concurrent entry of two hens in the same nest. Icken [20] reported percentages of double nest occupations that range between less than 1% up to 32%, in the case of flocks with different “hen-to-nest” ratios (*i.e.*, between 1:4.4 and 1:8.8). Furthermore, in this study, an improved FNB is described. This system is able to detect a double occupation of the nest, but to perform this task, it requires an additional mechanical part: a second tilting floor. The resulting system, therefore, has many mechanical components for each nest. In addition, the data collected for breeding programs may be less accurate, since normal laying behaviors could be discouraged by the presence of the trap devices [12].

Other systems, instead, do not try to avoid a double occupation of the nest through specific trap devices at the nest entrance. They only prevent more than one hen from occupying the nest at the same time by narrowing the nest [12]. In this way, the nest construction is simplified, but double nest occupations are not entirely avoided. Furthermore, these systems are not able to manage a double nest occupation when it occurs. The resulting dataset could, therefore, be incorrect.

The aim of the current study was to develop and test a nest usage “sensor”, based on imaging analysis, able to automatically detect a double nest occupation. Imaging analysis has never been used in this context, and it is an interesting approach, since it could allow the detection of the concurrent occupation of the nest without the use of any mechanical components, such as a double tilting floor. The nest construction for the recording systems, such as the FNB, could be simplified, while the recording systems that are not equipped with trap devices [12] and for which double nest occupations are not managed could be improved by the use of this sensor.

## 2. Experimental Section

The developed sensor was mainly composed of a commercial web-cam (HAMA AC-150) with a size of 50 mm × 15 mm and built-in LED lights. It was mounted on the top of the nest and controlled by a dedicated sensor software subroutine, which was developed using NI LabVIEW 8.2 (National Instruments^©^, Austin, TX, USA), NI Vision Acquisition Software 7.1 and the NI-IMAQ for USB cameras driver.

During the experiment, each image acquired by the web-cam from the nest interior was converted into a 2-dimensional array of colored pixels by the sensor software subroutine. Each colored pixel was compared to a “color background” (CB) threshold (*i.e.*, the nest interior, colored black for this purpose). From all of the comparisons carried out, the total amount of “colored pixels” (CP) was evaluated [28] and compared with the threshold “double nest occupation” (DNO). When the CP overcame the DNO threshold, a specific signal was sent by the sensor software subroutine to the experimental nesting system that automatically collected the hen performances and to which the sensor was connected. In the preliminary phase of the experiment, the DNO threshold was considered to be a value proportional to the mean surface of two hens (*i.e.*, the most restrictive case). In this way, the detection ability of the sensor also included cases in which more than two hens were in the nest.

### 2.1. Experimental Nesting System

An experimental nesting system was used to set-up and evaluate the developed sensor. It was designed as a single nest box (300 mm × 400 mm × 500 mm) without any trap device at the nest entrance. The floor (no bedding material) had an inclination of approximately 15° to allow the eggs to roll away immediately after laying, via an egg exit. Outside the nest, photo cells were positioned to detect laid eggs during their fall into the collection tube (which stored all of the daily production in the order of the laying). Photo cells had a global size of 55 mm × 55 mm × 72 mm, a diameter of 40 mm, a power supply of 12 V DC and a sampling frequency of 850 Hz ± 10%.

In the nest floor, a trapezoid-shaped antenna (250 mm × 400 mm × 20 mm) was embedded and coupled with a stationary ISO reader, BlueBox Gen2 LF, provided by Fasthink Srl. The reader was housed in a box of 110 mm × 140 mm × 60 mm, had a power supply of 24 V DC and an embedded RS232 protocol to be controlled by a PC. The resulting RFID system allowed the reading of transponders (ISO 11784/85 compliant), which were tightened to the hens' legs a day before the start of the experiment. According to published research [29,30], no evidence of a change in the hens' behavior was observed due to the wearing of the transponders.

### 2.2. Experimental Layout

**Figure 2.**
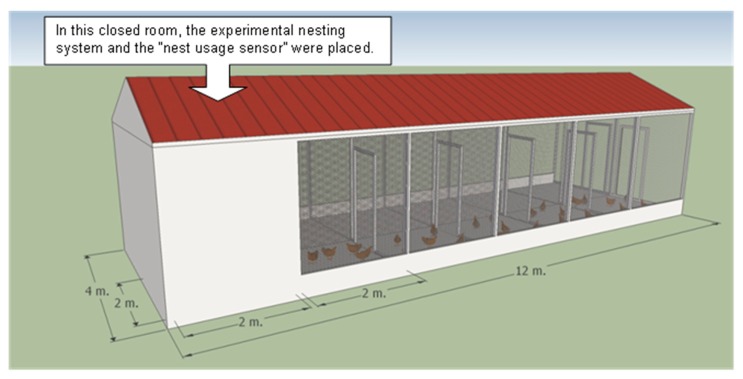
Drawing of the building where the experimental nesting system was installed. The dimensions of the building were 4 m × 12 m, and it included two closed rooms of 2 m × 2 m and five covered cages of 2 m × 2 m, to the right of each room. A part of the building was used for the experiment, involving a total floor space of 8 m^2^. It included a closed room and a covered cage, adjacent to the room, that provided 0.4 m^2^ of floor space per hen and appropriate perches.

**Figure 3.**
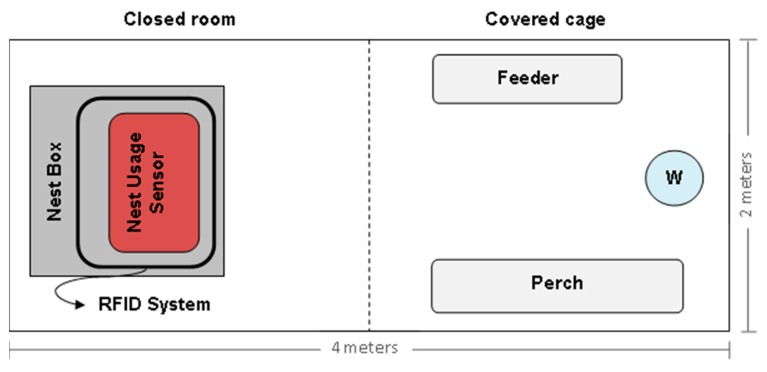
Diagram of the experimental building set-up. The positioning of the nest box, the nest usage sensor and of some main components is reported. The symbol “W” represents the water dispenser.

The experimental nesting system was installed in a separate building of a commercial laying farm, located in Lombardy (a region in Northern Italy), in which ISA (Institut de Sélection Animale) brown laying hens were reared. The building dimensions were 4 m × 12 m, comprised of two closed rooms, each 2 m × 2 m in size. To the right of each room, the building contained five covered cages of 2 m × 2 m. A part of the building was used for the experiment involving a total floor space of 8 m^2^. It included a closed room and a covered cage, adjacent to the room (Figures 2 and 3), that provided 0.4 m^2^ of floor space per hen and appropriate perches.

The percentage of double nest occupations is strongly influenced by the number of hens per nest [20]. Starting from the minimum standards for the protection of laying hens fixed by the EU (Council Directive 1999/74/EC), the value defined in Article 4.1.c (“all systems must be equipped in such a way that all laying hens have at least one nest for every seven hens”) was increased by *circa* 50%, in order to have a higher frequency of double nest occupations. As a consequence, among the total laying hens reared at the farm and already housed in the group, ten hens were randomly selected at the age of 18 weeks and housed a day before the start of the experiment in the experimental nesting system.

### 2.3. Experimental Design

Tests were carried out for six weeks. Data collected during the first two weeks were used to set-up the nest usage sensor, while the data collected in the following four weeks were used to evaluate its reliability.

During the sensor set-up phase, snapshots from the nest interior were triggered by the RFID-system (*i.e.*, a hen was considered in the nest when a transponder was read by the RFID reader). Acquired snapshots (*n* = 2127) were classified by a researcher, depending on the number of hens that were in the nest. This activity was necessary, because in this phase, double nest occupancies were not evaluated by the sensor. A subset of acquired snapshots (*n* = 400) was selected and analyzed through a specific release of the sensor software subroutine. The subset of snapshots was composed by 200 images of single nest occupations (randomly selected within 1239 snapshots, classified as a single nest occupation) and 200 images of double nest occupations (randomly selected within 411 snapshots, classified as a double nest occupation). This subroutine was developed to work off-line on folders of .bmp files. Three different CB (color background) thresholds were investigated corresponding to the three standard grey colors: 32-32-32, 64-64-64 and 96-96-96 (which are reported in the RGB color scale). For each CB threshold and selected snapshot, the corresponding number of CP (colored pixels) was evaluated. On each set of data obtained, the following analyses were performed:

- A one-way ANOVA procedure, in order to check for significance between the mean values of CP for the two cases investigated: Single or double nest occupation (*μ_S.O._ vs. μ_D.O._*).
- A ROC (receiver operating characteristic) analysis, in order to evaluate the sensitivity and specificity of the nest usage sensor in the detection of double nest occupations at different cut-off levels.

During the final step of the sensor set-up phase, a specific sensitivity was chosen (80%), and all the specificities for the different CB thresholds investigated were identified through the ROC curves built. For the following phase of the experiment, the CB threshold that showed the best result in terms of specificity was selected and used. Furthermore, the corresponding cut-off level was identified and used as the DNO threshold.

During the phase of the nest usage sensor evaluation, video recordings from the nest interior were acquired daily. Furthermore, data about laying performance and the behavior of hens were collected. In detail, each hen that entered the nest box was identified through its transponder. The laid egg was registered by photo cells when it rolled out from the nest box, and the egg-to-hen assignment was performed and then stored by the system. Double nest occupations were also detected by the nest usage sensor and recorded in the log file. At the end of the day, a researcher picked up the eggs from the nest collection tube, and the nest was then manually closed. Each egg was assigned to a specific animal according to the data provided by the experimental nesting system and properly marked. A mismatch between the data provided by the system and the sequence of eggs in the collection tube (due to missing eggs) was recorded. Finally, the video recording from the nest interior was evaluated in order to check the reliability of the nest usage sensor.

During testing, the lenses of the web-cam were checked daily and cleaned by a researcher to ensure that high quality images were acquired. The same was done for the photo cells to avoid possible reading mismatches.

## 3. Results

In the set-up phase of the nest usage sensor, different CB thresholds were investigated. The results obtained are reported in Table 1.

As shown, for each CB threshold evaluated, a difference was observed between the mean values of CP for the two possible cases: double or single nest occupation. In Table 2, the specificities of the nest usage sensor for each of the three CB thresholds investigated are reported, setting the sensitivity to be at least 80%.

As shown, the CB threshold that reached the highest specificity was the grey color, which corresponds to the value 64-64-64 (represented in the RGB color scale). On the basis of this result, in the next weeks of testing, the sensor software subroutine was set-up as follows: CB threshold equal to 64-64-64 (represented as 4210752 in the color integer scale) and DNO threshold equal to 20,920 colored pixels.

In the evaluation of the reliability of the nest usage sensor, the performances of the experimental nesting system were studied. During these tests, no changes in the average egg production were observed in comparison with the other hens of the farm. A total amount of 237 eggs was collected. The eggs laid in the nest were 228 (96.2% of the total eggs laid), while the eggs laid outside of the nest were nine (3.8%). The eggs immediately assigned to a laying hen of the trial group were 161 (70.6% of the eggs laid in the nest), while the eggs that were not assigned by the experimental nesting system were 67 (29.4%). The reasons for the egg-to-hen assignment failures were the following:

- Errors in the recording of the experimental data due to an interruption of the electrical power supply (1 egg, 0.4%);
- Missing eggs in the collection tube at the end of the day due to eggs caught in the nest (1 egg, 0.4%) or in the collection tube (3 eggs, 1.3%; most of the time, this was due to one egg being broken during its fall into the collection tube);
- Uncertain eggs within the sequences considered (62 eggs, 27.2%) because the sensor had automatically reported a double nest occupation.

In order to evaluate the accuracy of the nest usage sensor, video recordings from the nest interior were investigated. The results obtained are reported in Table 3.

As shown, the sensitivity and specificity reached by the nest usage sensor during the experiment were, respectively, 94.6% and 94.8%. Only three cases of double nest occupations occurred that were not detected by the sensor.

## 4. Discussion

The aim of the study was to develop a nest usage sensor able to detect a double nest occupation automatically. This target has driven the development and use of the sensor. The greater number of CPs over the DNO threshold in just one acquired image of the same double nest occupation was used to set-up the corresponding field in the log files. This use of the sensor explains the positive results obtained during the evaluation phase in a real scenario: 94.6% of double nest occupations occurring during ovipositions were correctly detected.

The developed sensor was shown to be sensitive to the low light and shadows that can be produced in the nest. These unfavorable conditions were, in general, well managed by the nest usage sensor through the set-up procedure performed and the algorithm design of the software that controlled the experimental nesting system. Nevertheless, some double nest occupations were not detected, while some singular occupations were wrongly classified by the sensor as double nest occupations. Double nest occupations that were not detected were mainly due to the low light conditions in the nest, which occurred during some ovipositions. Double nest occupations that were wrongly detected were instead mainly caused by shadows produced in the nest by the Sun. However, if the nesting system were installed in a bigger, closed building, equipped with artificial light, which would be more stable during the day, the conditions of low light and/or shadows in the nest could be avoided, and the accuracy of the sensor should be further improved.

In general, the results obtained showed that imaging analysis was an interesting approach to the aim of the present study. It allowed detecting the double nest occupations of hens automatically, during ovipositions, without the need for additional mechanical parts with respect to the nest. Consequently, this technical solution could simplify the nest construction for recording systems, like the FNB, which collect data about laying performance and the behavior of hens. Furthermore, the developed sensor could be easily added to other recording systems that are not equipped with trap devices [12]. This type of recording system would collect more accurate data, since the normal laying behavior of hens is left undisturbed, but it does not manage double nest occupancies. The developed sensor, if added to these systems, could allow for improving the accuracy of the data recorded, since double nest occupations would be managed. Finally, in this study, nest occupations of more than two hens were only detected by the sensor, but not discriminated. However, these cases could be easily managed by the sensor. Through a dedicated set-up, incremental DNO thresholds, representative of the presence of more than two hens in the nest, could be figured out, allowing the sensor to discriminate the exact number of hens that are in the nest. In a future development step of the sensor, in which more extensive experiments will be carried out in order to confirm the positive results obtained in this preliminary evaluation, this feature will probably be implemented.

In recent literature, other examples of technologies that allow collecting data about hens' behavior can be found [7,29]. These systems combine wireless sensor technology with GIS (Geographic Information Systems), and they allow collecting spatiotemporal data of hen movements in a well-defined housing system. Even though these systems can record data about nest usage, the technology investigated in this study could bring some advantages, if the main target is the recording of the laying performance of the hens. The identification of hens through RFID allows using smaller and cheaper sensors. If many nests are placed close to each other, the use of a floor-embedded antenna would permit an easier assignments between the hen and the corresponding visited nest. Egg-to-hen assignments can be made, and the resulting accuracy could be improved by the use of the proposed sensor, since double nest occupancies would be detected, automatically, for each nest. Finally, the technology investigated could be easily scaled up without specific problems related to the flock and/or facility sizes.

During the experiment, images from the nest usage sensor were converted into video recordings from the nest interior. In some cases, in studies on laying performance and the behavior of hens, dedicated video recording systems were used to acquire further information from the nest interior. For example, during the development of the FNB [8], four digital CCD cameras and a digital long-term recorder were used to evaluate the reliability of the nest occupancy times. In the present study, video recordings were used only to evaluate the accuracy of the developed sensor. Nevertheless, the same sensor could be easily used also to acquire other information from the nest interior without the need for additional and expensive systems dedicated to the recording of videos and/or images. In addition, the presence of at least one hen in the nest could be used as a trigger. The starting and ending of videos could be automatic, allowing for saving digital memory and time for evaluation. This trigger could be provided by the sensor as a further feature and be considered a practical improvement of the sensor when added to recording systems that acquire data about laying performance and the behavior of hens.

The nest usage sensor developed in this study was principally addressed to improve the accuracy of research systems that collect data about laying performance and the behavior of hens. This type of system has as its main target the collection of data about nest usage. The hens' behaviors, specific to free-range environments, are not generally discouraged. As a result, an improvement in the general knowledge about the hens' behaviors, in these alternative housing systems, can be reached with further positive practical implications also in terms of breeding selection, the definition of best practices in animals management, reviews in the design/set-up of housing systems, *etc.*, having as the final objective the improvement of the hens' welfare. Furthermore, the hardware components selected in the development of this sensor were shown to be easy to use, robust and inexpensive. Therefore, it is possible to suppose that this technological solution could be, in the future, scalded up to real production. The mechanical updates necessary to collect the laid eggs and the electronic infrastructure required to make the egg-to-hen assignments would be the main issues to solve. However, this improvement would be a real step forward in the egg production field, for both farmers and consumers. Farmers could achieve better management of the animals. As an example, they could identify hens that were less productive or hens that occupied a nest box for an extended period of time throughout the day, and in that case, they could decide to remove them from the flock. Consumers could obtain the detailed tracking information of the egg that, till now, has been limited to the egg production farm and the day of laying.

## 5. Conclusions

The results showed that the developed nest usage sensor allowed the detection of double nest occupations that occurred during the experiment automatically. The imaging analysis used in the development of the sensor resulted in being a useful technical solution that could simplify the nest construction for recording systems that collect data about laying performance and the behavior of hens, which are generally equipped with trap devices. Furthermore, the developed sensor could be easily added to other recording systems that are not equipped with trap devices. These systems would collect more accurate data, since the normal laying behavior of the hens is not disturbed by the presence of the trap devices. With the addition of the developed sensor, this type of system could allow for more precise data, since double nest occupations would be managed.

## Figures and Tables

**Figure 1.**
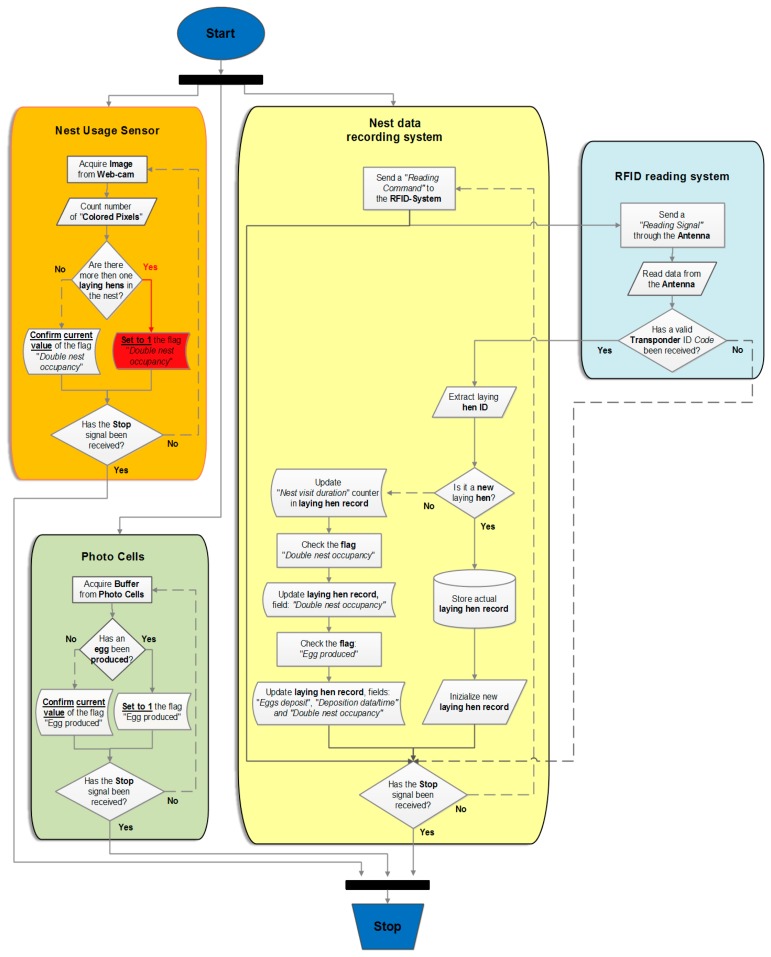
Application software flow diagram. The pathway in the case that a double nest occupation is detected is highlighted in red.

**Table 1.** Results of the one-way ANOVA procedure performed to check for significance between the mean values of colored pixels for the two cases investigated: single or double nest occupation. The statistical procedure was performed for each color background threshold investigated. The obtained means, SE and significance values are reported.

**Color Background Thresholds (RGB Scale)**	**Mean Value of Colored Pixels for Double Nest Occupations (***μ_D.O._* **Pixels)**	**Mean Value of Colored Pixels for Single Nest Occupations (***μ_S.O._* **Pixels)**	**Significance**
32-32-32	39,710 ± 519	24,274 ± 661	*p* < 0.01
64-64-64	27,829 ± 591	13,050 ± 537	*p* < 0.01
96-96-96	19,487 ± 590	7957 ± 422	*p* < 0.01

**Table 2.** Sensitivity and specificity values of the nest usage sensor evaluated for different color background (CB) thresholds.

**Color Background Thresholds (RGB Scale)**	**Sensitivity (%)**	**Specificity (%)**	**Corresponding Cut-off Level (Pixels)**
32-32-32	80	84	34,300
64-64-64	80	86	20,920
96-96-96	80	65	11,500

**Table 3.** The accuracy of the nest usage sensor in the detection of a double nest occupation during an oviposition.

	**Positive**	**Negative**	**Total**
True	53	163	216
False	9	3	12
Total	62	166	228
